# Health perception of a population-based study from 2006 to 2023 using multilevel modelling

**DOI:** 10.3389/fspor.2026.1702090

**Published:** 2026-02-05

**Authors:** Joni Marcio de Farias, Priscila Custódio Martins, Leandro de Oliveira Carpes, Ricardo Teixeira Quinaud

**Affiliations:** Postgraduate Program in Public Health – PPGSCol, University of the Extreme South of Santa Catarina – UNESC, Criciuma, Brazil

**Keywords:** BMI, education, epidemiology, exercise, temporal analysis

## Abstract

We aimed to analyze the variations in health perception among the Brazilian population, aggregated over 17 years of observation (2006–2023), considering individual and contextual characteristics. A total of 799,695 participants were included in the study, which had their data collected from the website of the Health Surveillance Secretariat, where the Surveillance System for Risk and Protective Factors for Chronic Diseases by Telephone Survey (Vigitel) is publicly available. Multilevel regression analysis was used to estimate the values of health perception among the Brazilian population, aggregated by year, region, BMI, education, sex, and exercise time. Based on the results, it was verified that over the years, health perception values decreased and there is a substantial disparity between Brazilian regions. Additionally, individuals with a healthy weight presented higher health perception values, individuals with more years of education had substantially higher health perception values and individuals who reported engaging in more than 30 min of exercise, when they exercised, had substantially higher health perception values. No differences in health perception were found between females and males. These findings highlight the need to prioritize actions to promote physical activity, increasing the availability, access and quality of public places, preferably with assistance/exercise prescription in these places and free of charge. It is essential to assess past initiatives and integrate efforts to optimize future strategies and to identify strategies that reduce inequities in access to health-promoting environment.

## Introduction

Population-based epidemiological studies are defined as research that covers a representative sample of the population and is important for identifying the distribution of exposure and disease, as well as the conditions that influence the dynamics of health risk patterns in a given community (1). Health self-assessment has been used in population-based studies to assist in developing strategies and public policies aimed at improving the population's health by promoting active living and well-being such as parks and green spaces (2, 3). Self-rated health is considered a simple and easily applicable measure and is associated with clinical indicators of morbidity and mortality (4). Furthermore, self-rated health can serve as an excellent marker of differences among population subgroups (5).

Health perception encompasses the positive or negative evaluation of an individual's general health status and integrates biological, mental, social, and functional aspects, including individual and cultural beliefs and health behaviors (6). Researchers have identified that individuals who report “poor” health have twice the risk of all-cause mortality compared to those who report “excellent” health (5, 7).

Health perception can be influenced by various factors, such as sex, age, and level of education (8–10). In Brazil, population-based studies have identified an association between unfavorable socioeconomic conditions and specific demographic characteristics with negative health perceptions (9, 11). However, given the difficulty of collecting data from different time points, especially over periods longer than 15 years, interpretations and decisions are often based on simple cross-sectional time point studies. Thus, this study aimed to analyze variations in health perception among the Brazilian population, aggregated over 17 years of observation (2006–2023), considering both individual and contextual characteristics.

## Methods

The present study is characterized as a temporal study. Thus, all years were analyzed (from 2006 to 2023). Data were collected on March 26, 2024, from the website of the Health Surveillance Secretariat, where the Surveillance System for Risk and Protective Factors for Chronic Diseases by Telephone Survey (Vigitel) is publicly available (https://svs.aids.gov.br/download/Vigitel/). According to the Ministry of Health, “Vigitel is a telephone survey conducted annually by the Brazilian Ministry of Health since 2006, aiming to monitor the main risk and protective factors for chronic non-communicable diseases. In each edition of Vigitel, a simple random sample of adults aged ≥18 years living in households with at least one landline phone in the 26 Brazilian state capitals and the Federal District is surveyed. The 2022 survey was not available on the website. However, data were collected in that year and included in the 2021 survey.

The questionnaire includes 83 questions covering demographic and socioeconomic data, dietary patterns, lifestyle habits, anthropometric data, self-assessment of health status, and self-reported morbidity. The question used in the present study to collect the information about health perception was “How would you classify your state of health?”. In this study, the sociodemographic variables considered were: region of residence (Central-West, Northeast, North, Southeast, South); sex (men, women); education level (0–4 years of schooling, 5–8 years, 9–11 years, 12 or more years of schooling); physical activity (no exercise, ≤29 min, and ≥30 min); and self-assessment of health status. Participants rated their health perception on a scale from one (very good) to five (very bad). To facilitate the interpretation, we reversed the values on Table 1.

**Table 1:** Absolute frequencies, relative frequencies and health perception values in relation to year, region, BMI, level of education, sex and time of exercise.

Variable	Sample (*n* = 799,695)	Percentage (%)	Health perception (SD*)
Year			
2006	52,644	6.6	3.75 (0.77)
2007	55,510	6.9	3.75 (0.77)
2008	54,223	6.8	3.81 (0.82)
2009	54,215	6.8	3.82 (0.82)
2010	54,161	6.8	3.81 (0.82)
2011	53,826	6.7	3.79 (0.83)
2012	44,996	5.6	3.78 (0.83)
2013	51,927	6.5	3.73 (0.80)
2014	40,233	5.0	3.72 (0.80)
2015	53,273	6.7	3.74 (0.82)
2016	52,971	6.6	3.79 (0.83)
2017	52,550	6.6	3.78 (0.81)
2018	51,864	6.5	3.75 (0.82)
2019	51,881	6.5	3.74 (0.83)
2020	26,912	3.4	3.78 (0.81)
2021	21,136	2.6	3.73 (0.85)
2022	5,790	0.7	3.73 (0.85)
2023	21,583	2.7	3.69 (0.86)
Region			
Midwest	119,704	15.0	3.83 (0.82)
North	268,609	25.2	3.74 (0.81)
Northeast	201,739	33.6	3.69 (0.80)
South	119,944	11.2	3.89 (0.83)
Southeast	89,699	15.0	3.83 (0,82)
BMI			
Underweight	26,783	3.3	3.71 (0.84)
Healthy weight	350,087	43.8	3.88 (0.79)
Overweight	280,880	35.1	3.76 (0.80)
Obesity	141,945	17.7	3.50 (0.84)
Education			
0–8 years	225,098	28.1	3.49 (0.84)
9–11 years	295,152	36.9	3.76 (0.78)
≥ 12 years	279,445	34.9	3.99 (0.76)
Sex			
Female	496,061	62.0	3.71 (0.83)
Male	303,634	38.0	3.86 (0.78)
Exercise			
No exercise	409,953	51.3	3.59 (0.83)
≤ 29 min	43,500	5.4	3.84 (0.77)
≥ 30 min	346,242	43.3	3.97 (0.76)

* SD, standard deviation.

A multilevel regression model was used to analyze variations in participants’ responses regarding health perception when grouped by year (2006–2023), region (Midwest, North, Northeast, South, and Southeast), BMI (underweight, healthy weight, overweight, and obesity), education (0–8 years, 9–11 years, and ≥12 years), sex (female and male), and exercise duration (no exercise, ≤29 min, and ≥30 min). Multilevel analysis provides a more robust, reliable, and flexible alternative by accounting for the hierarchical structure of the data as well as the information available between groups. This analysis estimates participants’ values based on individual and group characteristics (12).

A varying intercept model was employed, allowing the intercept to vary across participants’ responses (Level 1), nested within groups (Level 2; year, region, BMI, education, sex, and exercise duration), providing a more precise estimate. Estimates were derived using the maximum likelihood method with the “lme4” package (13) in the R statistical language (14). The data were standardized, and results are presented as estimates with 95% confidence intervals.

## Results

A total of 806,169 participants had their data collected by Vigitel. However, 6,474 participants were excluded from this study because they did not respond to the survey on health perception. Thus, the final sample of the present study comprised 799,695 participants. Notably, only the data from 2022 showed a lower number of participants compared to other years, as explained in the methodology section. Additionally, a decline in data collection after 2020 was observed. Regarding regional distribution, the Southeast had the lowest participation rate despite being the most populated region of Brazil. Most participants had a healthy weight based on BMI. However, when overweight and obese individuals were combined, they constituted the majority of the sample. Lastly, participants had varying levels of education, the majority were women, and most did not engage in physical exercise (Table 1).

Table 2 presents the estimates and 95% confidence intervals for health perception considering year, region, BMI, education, sex, and exercise duration. Over the years, health perception values decreased. For example, values from 2007 to 2010 were higher and substantially different from those of 2021, 2022, and 2023, the period following the COVID-19 pandemic. However, when comparing the values from 2007 to 2010 with other years, a substantial decrease was also observed.

**Table 2:** Estimate and confidence interval (95%) of the health perception considering year, region, BMI, education, sex and time of exercise.

Variable	Estimate (95% confidence interval)
Year	
2006	0.00 (−0.02–0.02)
2007	0.06 (0.04–0.08)
2008	0.08 (0.06–0.10)
2009	0.08 (0.06–0.11)
2010	0.08 (0.05–0.10)
2011	0.05 (0.03–0.08)
2012	0.03 (0.01–0.05)
2013	−0.03 (−0.05 to −0.01)
2014	−0.04 (−0.06 to −0.01)
2015	−0.03 (−0.05–0.00)
2016	0.03 (0.01–0.05)
2017	0.02 (0.00–0.04)
2018	−0.02 (−0.04–0.00)
2019	−0.02–0.04–0.00)
2020	0.03 (0.00–0.05)
2021	−0.01 (−0.03–0.02)
2022	0.00 (−0.03–0.03)
2023	−0.07 (−0.10 to −0.05)
Region	
Midwest	0.05 (−0.03–0.13)
North	−0.07 (−0.15–0.01)
Northeast	−0.11 (−0.19 to −0.02)
South	0.11 (0.03–0.19)
Southeast	0.08 (0.00–0.16)
BMI	
Underweight	0.08 (−0.07–0.22)
Healthy weight	0.17 (0.02–0.32)
Overweight	0.03 (−0.12–0.17)
Obesity	−0.22 (−0.36 to −0.07)
Education	
0–8 years	−0.24 (−0.45 to −0.04)
9–11 years	0.03 (−0.18–0.24)
≥ 12 years	0.26 (0.05–0.46)
Sex	
Female	−0.06 (−0.19–0.07)
Male	0.09 (−0.05–0.22)
Exercise	
No exercise	−0.16 (−0.34–0.01)
≤ 29 min	0.03 (−0.14–0.20)
≥ 30 min	0.18 (0.01–0.35)

Regarding regional differences, we identified a substantial disparity in health perception between individuals living in the Northeast, who had the lowest values, and those in the South, who had the highest values. In terms of BMI, individuals with a healthy weight exhibited higher health perception values, with a substantial difference compared to obese individuals. Similarly, when comparing education levels, individuals with more years of education had substantially higher health perception values than those with fewer years of education. Lastly, individuals who reported engaging in more than 30 min of exercise when they exercised had substantially higher health perception values compared to those who did not exercise. No differences in health perception were found between females and males.

## Discussion

This temporal study, based on 17 years of cross-sectional data, demonstrated that individuals with higher education levels, a healthy weight, and regular physical activity tend to have a higher perception of health. The significant regional disparities in Brazil point to the necessity of reassessing policies implemented in certain areas, as well as fostering collaboration between states to share successful strategies for improving public service delivery. These findings highlight the urgency of reviewing and improving health and education policies to enhance their effectiveness.

Brazil is characterized by marked regional heterogeneity in socioeconomic development, health infrastructure, education, and access to public services, and regional income inequality is a persistent feature of Brazilian socioeconomic development. The North and Northeast regions, which consistently presented lower self-rated health in the present study, are historically marked by lower income levels, reduced educational attainment, limited access to healthcare services, and fewer opportunities for health-promoting behaviors such as leisure-time physical activity (15). These structural disadvantages likely contribute to poorer health perception by increasing exposure to chronic conditions, reducing preventive healthcare utilization, and limiting access to safe and adequate environments for physical activity (16). In contrast, the South and Southeast regions benefit from higher socioeconomic indicators, more consolidated health systems, and greater availability of public and private health-related resources, which may partially explain their more favorable health perception profiles (17). These findings reinforce the need to reassess regional health and education policies and to promote interregional cooperation aimed at reducing internal structural inequalities.

Similar findings were reported in a population-based study conducted between 2002 and 2005 in 18 Brazilian capitals, involving 26,424 individuals over 15 years of age. The results showed that negative health perception was more prevalent among individuals with lower education levels and those living in the North and Northeast regions across all age groups (18). Additionally, South and Southeast regions have the highest average levels of schooling, with a greater proportion of adults who have completed high school and higher education, while the North and Northeast regions have higher rates of functional illiteracy and lower school retention, reflecting historical trajectories of lower educational investment and greater social vulnerability.

Likewise, a study analyzing data from household surveys conducted between 1998 and 2013 found that the North and Northeast regions exhibited the worst health indicators, which were associated with a higher prevalence of negative health perception (19). Regarding access to health services, although the Brazilian public system, organized by the Unified Health System (SUS), has national coverage, marked regional disparities persist, with greater availability of specialized services, health professionals, and hospital infrastructure in the South and Southeast regions, contrasting with greater difficulties in access, long distances, and lower healthcare provision in the North and Northeast.

The indicators investigated included education level, prevalence of chronic diseases such as hypertension and diabetes, and the number of medical consultations within the Brazilian public health system [Sistema Único de Saúde (SUS)]. Furthermore, data from the World Health Survey indicated that individuals with lower education levels had poorer health assessments and made less use of healthcare services (20). These findings highlight the need for public policies aimed at ensuring equity in healthcare access and improving education levels. These structural inequalities directly impact health-related behaviors, since population studies indicate a higher prevalence of regular physical activity during leisure time in the South and Southeast regions, associated with higher education levels, income, and availability of adequate spaces, while the North and Northeast regions show lower levels of physical activity during leisure time and greater dependence on occupational efforts or active commuting, often in less favorable contexts. Higher levels of education provide greater access to health information, fostering the adoption of healthy habits and expanding employment opportunities, thereby ensuring better access to healthcare services throughout life.

In the present study, individuals who engaged in at least 30 min of physical exercise showed a more positive perception of their own health. This association between physical activity and self-perceived health is corroborated by evidence in various adult populations and contexts. A population-based study in the Brazilian Amazon demonstrated that physically active adults reported better self-perceived health and fewer cardiovascular risk factors, highlighting how regular daily movement relates to better perceived health, even in socioeconomically disadvantaged contexts (21). Complementing these findings, a nationwide cross-sectional study carried out in 2019 with 25,785 Brazilian adults and older adults from all state capitals showed a clear dose–response relationship between leisure-time walking and positive self-rated health. Individuals who reported walking between 150 and 299 min per week and those who accumulated at least 300 min per week were 28% and 52% more likely, respectively, to report positive health perception compared with those walking less than 150 min per week (22). International guidelines, such as those from the World Health Organization (23), American Heart Association (24), and for the Brazilian population (25), recommend that adults engage in at least 150–300 min of moderate-intensity physical activity per week (30 min for day) to improved mental health, reduced risk of chronic diseases, and enhanced quality of life. This convergence between epidemiological evidence and guideline recommendations strengthens the argument for public policies that prioritize physical activity promotion, particularly through accessible and structured programs in public spaces.

In the present study, self-perceived health varied according to BMI status, with obese individuals reporting lower health perception values compared to those with normal weight. A longitudinal study (1998–2015) found that higher BMI levels were associated with poorer self-rated health among middle-aged participants. However, among older adults, worse self-rate health was linked to a more pronounced decline in BMI (26). The BMI trajectories are linked to self-rate health, though the nature of this association may vary with age. In older adults, the decrease in BMI is primarily related to the natural aging process, which leads to the progressive loss of muscle mass. Even when body weight remains stable, changes in body composition can occur, with an increase in fat and a loss of muscle, negatively impacting overall health (27). This alteration in body composition directly affects the elderly's perception of their health, as they may report increased fatigue, reduced mobility, and decreased independence in daily activities, all contributing to a more negative view of their well-being (28).

This study has some limitations, such as the data was based on self-reports, decline of responses after 2020, no varying slopes were tested and a limited number of variables in the model. However, it also presents several strengths, including the use of a temporal analysis over 15 years, population-based sample representative of Brazilian capitals, the application of a validated questionnaire developed by Vigitel and the use of multilevel regression analysis for data interpretation. Especially when analyzed together, the link between lower levels of education, more limited access to health services, and fewer opportunities for regular physical activity contributes to the maintenance of regional health inequities, reinforcing the need for intersectoral policies that integrate education, health, and the promotion of active lifestyles, with a priority focus on historically more vulnerable regions.

## Conclusion

As time of exercise presented influence on health perception, the development of natural environments for physical exercise is encouraged. There is a need to prioritize actions to promote physical activity, increasing the availability, access and quality of public places, preferably with assistance/exercise prescription in these places and free of charge. We also seek to identify strategies that reduce inequities in access to health-promoting environments, particularly for vulnerable populations such as children, the elderly, and socioeconomically disadvantaged groups. It is essential to assess past initiatives and integrate efforts to optimize future strategies.

The severe discrepancies observed between Brazil's regions indicate that policies implemented over the years in certain areas require reassessment. As Brazil operates under a federalist system, states from different regions should collaborate and share successful strategies to improve public service delivery. Regarding the trends in health perception over the 17-year period, the decline observed during and after the COVID-19 pandemic is understandable. Lastly, as education level and BMI also influence health perception, public health centers could implement orientation programs to help the community better understand healthy habits and promote health behavior change.

## Data Availability

Publicly available datasets were analyzed in this study. This data can be found here: https://svs.aids.gov.br/download/Vigitel/).
